# Damage tolerance of nuclear graphite at elevated temperatures

**DOI:** 10.1038/ncomms15942

**Published:** 2017-06-30

**Authors:** Dong Liu, Bernd Gludovatz, Harold S. Barnard, Martin Kuball, Robert O. Ritchie

**Affiliations:** 1 Department of Materials, University of Oxford, Oxford OX1 3PH, UK; 2 Materials Sciences Division, Lawrence Berkeley National Laboratory, Berkeley, California 94720, USA; 3 Advanced Light Source, Lawrence Berkeley National Laboratory, Berkeley, California 94720, USA; 4 Center for Device Thermography and Reliability, H.H. Wills Physics Laboratory, University of Bristol, Bristol BS8 1TL, UK; 5 Department of Materials Science and Engineering, University of California, 324 Hearst Mining Building, MC 1760, Berkeley, California 94720, USA

## Abstract

Nuclear-grade graphite is a critically important high-temperature structural material for current and potentially next generation of fission reactors worldwide. It is imperative to understand its damage-tolerant behaviour and to discern the mechanisms of damage evolution under in-service conditions. Here we perform *in situ* mechanical testing with synchrotron X-ray computed micro-tomography at temperatures between ambient and 1,000 °C on a nuclear-grade Gilsocarbon graphite. We find that both the strength and fracture toughness of this graphite are improved at elevated temperature. Whereas this behaviour is consistent with observations of the closure of microcracks formed parallel to the covalent-*sp*^2^-bonded graphene layers at higher temperatures, which accommodate the more than tenfold larger thermal expansion perpendicular to these layers, we attribute the elevation in strength and toughness primarily to changes in the residual stress state at 800–1,000 °C, specifically to the reduction in significant levels of residual tensile stresses in the graphite that are ‘frozen-in’ following processing.

## Abstract (web summary)

Nuclear-grade graphite is an important high-temperature structural material for fission reactors. Here, the authors perform simultaneous X-ray tomography and mechanical testing on a nuclear-grade graphite, finding simultaneous improvement of strength and toughness at elevated temperatures which they attribute primarily to reduction of residual tensile stresses in the as-made material.

Nuclear-grade graphites are used as a ‘neutron moderator’ in roughly one-fifth of the world’s currently operating nuclear reactors. These reactors rely on the high scattering cross-section of graphite to moderate the energies of fast neutrons, converting them into thermal neutrons to sustain the uranium-235 fission chain reaction^1^,^2^. Graphite also serves as a non-replaceable structural component in these reactor cores^3^,^4^, which must remain stable over the lifetime of the reactor while operating at elevated temperatures. Presently, UK Advanced Gas-Cooled Reactors (AGR) are designed to operate at temperatures of 420–650 °C, but future Gen-IV designs, such as very high temperature reactors, will potentially operate as high as 1,000 °C with a graphite core^5^,^6^. Despite graphite’s use in such safety-critical applications, the nano- and micro-structure of nuclear graphite has an extremely high defect population that spans multiple length scales, leading to physical properties that are not completely understood. As the mechanical behaviour of materials is dominated by the presence of defects, we seek here to fully characterize the strength and toughness of this material at typical in-service temperatures between ambient and 1,000 °C.

In particular, Gilsocarbon graphite is currently used in the operating UK AGRs and is a representative material for future nuclear graphites designed for Gen-IV reactors. Gilsocarbon graphite is a moulded, medium-grained, near-isotropic (anisotropy ratio of 1:1.1) GCMB grade polygranular nuclear graphite with the bulk elastic modulus in the range of 10–11 GPa at ambient temperatures^7^,^8^. Its structure comprises spherical filler particles of Gilsonite (a naturally occurring solid hydrocarbon bitumen), about 500 μm in diameter, in a binder matrix made from the finer fractions of the coke flour and coal tar pitch (see Supplementary Fig. 1). The filler particles, which originate from the coarse-milled Gilsonite coke grains and make up some 70–80% of the total weight, consist of small contiguous crystallites that are misaligned to form a circumferential pattern that contributes substantially to the near-isotropic properties of this grade of graphite.

Although displaying relatively little anisotropy, the strength properties of Gilsocarbon are very sensitive to sample size, owing to its high multi-scale defect population. Depending on the orientation relative to the moulding compression direction, the ambient-temperature (macro-scale) flexural strength in millimetre- to centimetre-sized samples is ∼26–27 MPa (ref. 8), whereas micro-mechanical tests on the same material, using 2 × 2 × 12 μm-sized bend samples, give values of the flexural strength as high as ∼1,000 MPa (ref. 9), the latter micrometre-scale samples sizes excluding effects of the macro- and micro-size pores. Such a marked size dependence of the strength properties is not that unusual for quasi-brittle materials such as graphite that possess multiple length-scale defect populations; further details are provided in Supplementary Fig. 2. In terms of the fracture characteristics of graphites, there have been numerous studies performed, but these have primarily been focused on ambient temperature behaviour^1^,^2^,^3^,^4^,^6^,^7^,^10^.

The defect population of Gilsocarbon is both large and varied, and acts to influence the behaviour of the material over its operating temperature range. The filler particles represent one type of defect, as they are invariably cracked along their basal (*sp*^2^-bonded graphene) planes as a consequence of the weak bonding between these planes and the volumetric shrinkage during calcination (∼1,300 °C) to form mostly closed lenticular porosity. The porosity in the binder phase includes large circular open pores (usually several hundred micrometres in diameter), pore runs resulting from the bubble percolation of volatile gases during the carbonization baking stage (∼800 °C) and micrometre-sized pores formed during the impregnation of liquid pitch. In addition, lenticular cracks form, in both filler particles and the binder matrix, parallel to the basal planes during cooling down from the final graphitization temperature (typically 2,700–3,000 °C) due to anisotropic contraction of the hexagonal unit cells (thermal expansion coefficients are ∼27 × 10^−6^ K^−1^ perpendicular to the basal plane and approximately −1.5 × 10^−6^ K^−1^ along the parallel direction)^11^,^12^,^13^. These lenticular cracks, termed Mrozowski cracks, are typically sized from several nanometres to several hundreds of nanometres in width with lengths tens of times larger (Supplementary Fig. 1)^12^,^13^. Many of these cracks that form nominally parallel to the basal planes tend to close upon heating^12^, similar to those reported for highly oriented pyrolytic graphites based on *in situ* transmission electron microscopy (TEM) investigations at, and above, 800 °C (refs 12, 14).

To investigate how such multiscale defect populations affect mechanical properties at elevated temperatures, here we characterize the (macro-scale) strength and fracture toughness of this Gilsocarbon graphite using three-point bend samples at a temperature range between 20 °C and 1,000 °C. These experiments are performed at a synchrotron X-ray beamline with the samples mounted in a high-temperature test cell (hot cell, see Supplementary Fig. 3). This setup allows real-time three-dimensional (3D) computed micro-tomography, coupled with post-mortem digital volume correlation (DVC), to image and quantify the micro-scale damage and fracture processes. We find that, contrary to the behaviour observed in the vast majority of materials, both the strength and fracture toughness of nuclear graphite are improved at elevated temperature. We attribute this elevation in strength and toughness to the change of residual stresses accompanied by the closure of nano-scale cracks at temperature.

## Results

### High-temperature strength and fracture toughness *J*_R_-curves

**Figure 1:**
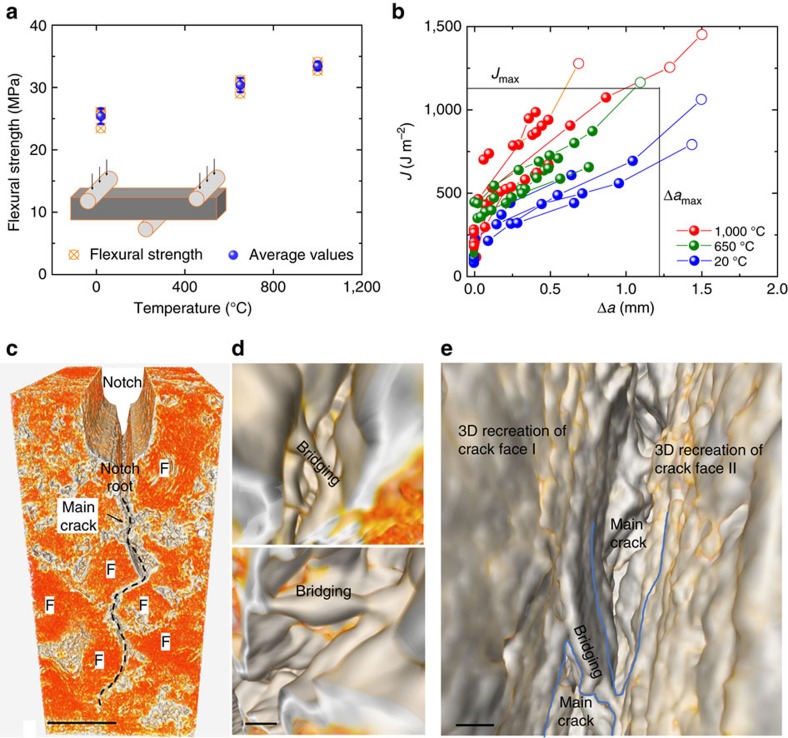
Strength and toughness of Gilsocarbon graphite as a function of temperature. (**a**) The macro-scale flexural strength (measured on 3–4 mm-sized bend specimens) increases with temperature from ∼25 MPa at 20 °C to ∼32 MPa at 1,000 °C and (**b**) the corresponding fracture toughness also increases, in terms of *J*_R_(Δ*a*) crack-resistance curves. (It is noteworthy that the open data points on the R-curves in **b** are outside the maximum *J* and Δ*a* limits for the size of our specimens, as prescribed by the ASTM E1820 standard^41^; these data points are not included in the analysis. Owing to the size of the samples, these data points represent a condition of large-scale bridging, where the extent of crack-tip shielding in the wake of the crack tip is no longer small compared with the in-plane dimensions of crack size and the remaining uncracked ligament). (**c**–**e**) Reconstructed tomographic 3D volume images of Gilsocarbon under load at 1,000 °C, showing (**c**) a typical crack originating from the notch root deflected around filler particles (labelled F); scale bar, 400 μm, (**d**) individual bridging sites formed during the growth of the crack; scale bar, 10 μm, and (**e**) an example of a larger volume of material forming a bridging region in the path of the advancing main crack; scale bar, 20 μm. False colour used in **c**–**e** to highlight various features.

Our experiments afforded two principal findings with regard to Gilsocarbon graphite’s mechanical properties. First, with macro-scale tests, the maximum flexural strength of the graphite was found to be about 30% higher at 1,000 °C than at ambient temperature, specifically rising from ∼25 MPa at 20 °C to ∼32 MPa at 1,000 °C (Fig. 1a). Second, using *in situ* tomography to quantify the 3D crack geometry, full nonlinear-elastic fracture-mechanics based *J*_R_(Δ*a*) crack-resistance curves (R-curves) were derived at 20, 650 and 1,000 °C, as shown in Fig. 1b in terms of *J* as a function of crack extension Δ*a*; this revealed a corresponding increase in the fracture toughness of the nuclear graphite with temperature. Specifically, mean *K*_Ic_ crack-initiation toughness values increase approximately twofold from ∼1–1.5 MPa✓m at 20 °C to ∼2–3 MPa✓m at 1,000 °C, when back-calculated in terms of stress intensities (see Methods). The steepness of the R-curves, representative of the crack-growth toughness, was also observed to increase at the higher temperatures. The full R-curves, re-plotted in terms of stress-intensity factors, are shown in Supplementary Fig. 4.

This elevation in the strength and fracture toughness at increasing temperature is a highly unusual characteristic of a material. Although this has been reported for some earlier nuclear-grade graphites in certain previous studies^15^,^16^,^17^, to our knowledge, this has never been satisfactorily explained in mechanistic terms, nor have the individual toughening mechanisms associated with crack initiation and crack growth been partitioned, as shown by this first reported R-curve analysis for Gilsocarbon graphite at 1,000 °C (Fig. 1b).

### Extrinsic toughening mechanisms

To provide such an explanation of this unusual elevated-temperature behaviour in nuclear graphite, we performed a comprehensive examination of the tomographic images and identified several extrinsic toughening mechanisms at both ambient and high temperatures which contribute to the rising R-curve behaviour. (Note here that resistance to fracture can be considered as a mutual competition between two classes of toughening mechanisms: intrinsic mechanisms, which represent material’s inherent resistance to microstructural damage mechanisms that operate ahead of the crack tip, plasticity or, more generally, inelasticity, being the dominant contributor, and extrinsic mechanisms, which act to ‘shield’ the crack from the applied driving force and operate principally in the wake of the crack tip. Extrinsic toughening is only effective in developing crack-growth toughness, as demonstrated by a rising R-curve; these mechanisms have little to no influence on the crack-initiation toughness^18^.)

**Figure 2:**
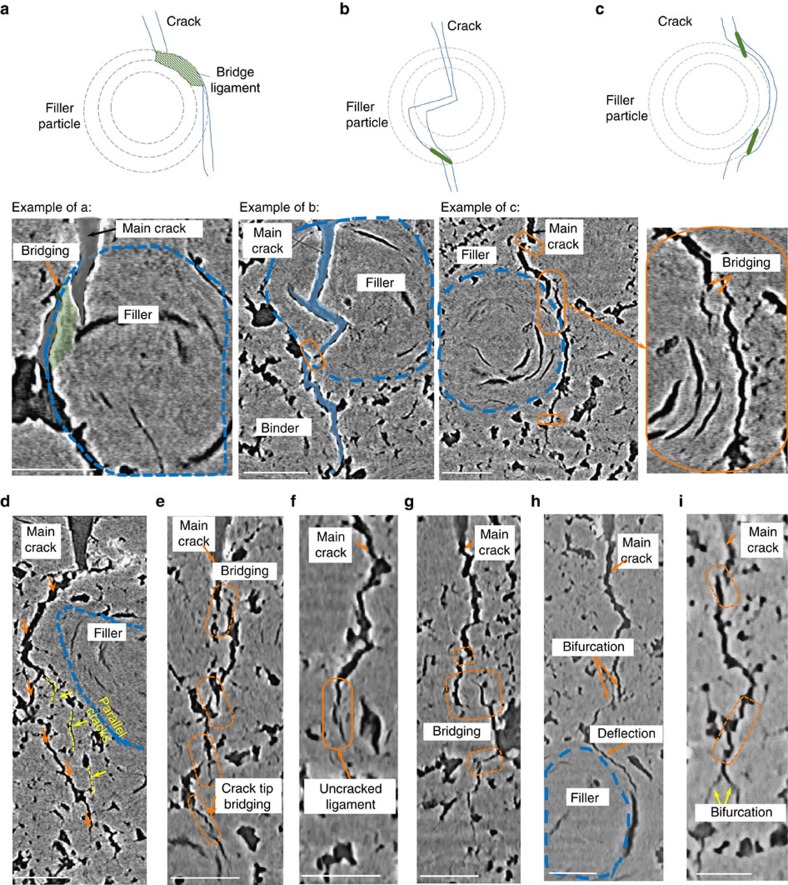
Computed tomography shows generation of extrinsic toughening mechanisms. In Gilsocarbon graphite, crack deflection and crack bridging occurs at 20 °C when the main crack interacts with a filler particle in three ways: (**a**) as the main crack grows through the side of a filler particle, the material between the pores around the particle and circumferential crack inside the particle tend to form a bridge site; (**b**) the crack enters through the centre of a particle and is deflected by the central calcination cracks such that bridges form when it exits the particle to join the matrix; (**c**) the crack misses the main part of the particle but bridges still form around the filler particle due to the change in crack path; (**d**) example of parallel crack coexisting with the main crack at 20 °C. Examples at 1,000 °C of (**e**) the discontinuous main crack interrupted by various bridging sites and increased number of small bridges at the crack tip, combined with uncracked-ligament shielding; (**f**) uncracked-ligament bridging near the crack tip; (**g**) the combination of very large ligament bridging and very small ones as the crack path changes direction; (**h**) crack deflection at filler particles; and (**i**) with consequent bifurcation occurring at the crack tip. All scale bars, 100 μm.

As seen in Fig. 2, many of the filler particles are cracked along their folded graphene planes; interactions between the main crack path and these particles lead to gross deflection and twist of the crack front as the crack essentially circumvents/passes each particle. Such crack deflections act to increase the contribution from extrinsic toughening (Supplementary Fig. 5). Constrained microcracking around the vicinity of the main crack, uncracked-ligament bridging in the crack wake and bifurcation at the crack tip^18^,^19^ are also observed at all three temperatures (Supplementary Fig. 6). At room temperature, the generation of bridging sites in the form of microcracks, located ahead of the main crack, acts to create such uncracked-ligament crack bridges, which in turn result in a change in the propagation direction; both serve as potent extrinsic toughening mechanisms. Essentially, crack growth in graphite can be envisaged as a process of breaking a network of bridging ligaments resulting in a meandering crack path. There appears to be two prime mechanisms for the formation of crack bridges at ambient temperatures: one, as stated above, is when the crack passes a filler particle; the other is when the crack enters a different phase, for example, when the crack propagates from a pore to a solid region; both processes act to cause the direction of the crack path to change. Specifically, there are several main consequences to form bridging sites when a moving crack interact with a filler particle from one side (Fig. 2a), through the middle (Fig. 2b), and around the particle (Fig. 2c). Such bridging sites, although naturally formed close to the crack tip, are retained as part of a bridging zone in the crack wake, in some cases spanning up several hundred micrometres. Moreover, with the preponderance of microcracks and pores in the microstructure of the graphite, several parallel cracks often co-exist (Fig. 2d), which can further diminish the driving force for (fatal) crack extension.

At higher temperatures, conversely, there appears to be a reduction in the micro-crack density at 1,000 °C, as compared with that at ambient temperatures (Supplementary Fig. 6). Specifically, the circumferential lenticular cracks within the filler particles tend to become smaller in size when the graphite is heated up to 1,000 °C (Supplementary Fig. 7). However, more bridging sites are observed at 1,000 °C and they are more densely distributed along the crack path (Fig. 2e), which certainly contributes to extrinsic toughening and hence to the increasing steepness of the R-curves, that is, higher crack-growth toughness, at the higher temperatures. In particular, there is more evidence of uncracked-ligament bridging (Fig. 2f,g) than is seen at lower temperatures (in some samples, there is an increase by ∼50% in bridge density, as compared with that at 20 °C); although many of these bridges are quite narrow (≤5 μm in size), they extend further into the crack wake than the less numerous ones seen at lower temperatures. Certainly, the deflection of the crack by filler particles is still active at high temperatures (Fig. 2h). In particular, at 1,000 °C, there is more evidence of bifurcation of the crack tip (Fig. 2h,i and Supplementary Fig. 6). Interactions between the main crack and filler particles, pores and existing defects still contribute to the deflection of the crack path, which further provides extrinsic toughening, but there is distinctly less microcracking involved in such events at high temperatures (Supplementary Fig. 6). In addition, due to the presence of larger ligament bridges along the crack length (crack bridges are either very large, ∼100 μm, or very small, ≤5 μm, and regularly spaced along the crack path), the main crack path appears almost discontinuous when viewed in a two-dimensional slice (Fig. 2e,g).

### Visualization and quantification of cracks and strains

**Figure 3:**
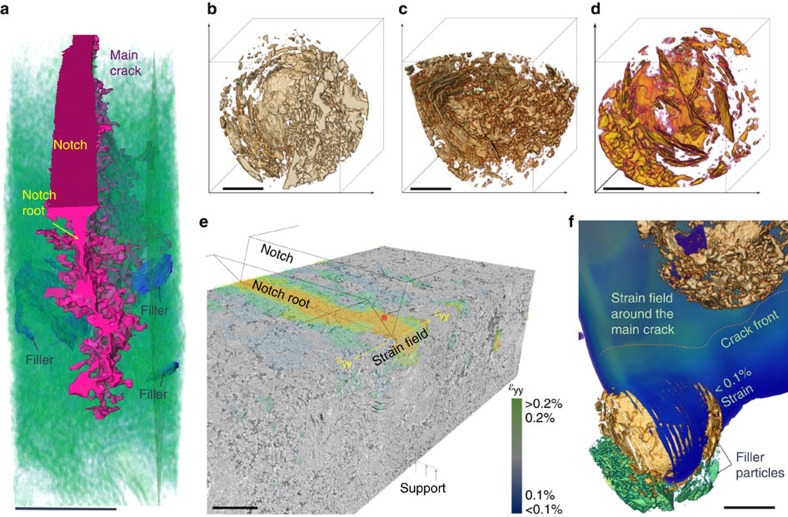
Tomographic reconstructions of fracture in nuclear graphite at 1000 °C. (**a**) Fracture at 1,000 °C showing a complex geometry (planar section at top is the notch mouth). The pore structure of the filler particles is shown to demonstrate their location to the crack path. Scale bar, 1 mm; (**b**–**d**) typical filler particles, colour representing pores within long and narrow circular layers (scale bar, 100 μm); (**e**) 3D *ε*_yy_ strain field, averaged over ∼100 μm^3^ volume, close to the notch tip at 90% of crack-extension load, overlaid onto a 3D volume of graphite in bending (material down to the notch depth removed to reveal notch-root strains). Scale bar, 750 μm; (**f**) DVC calculation of 3D maximum principal strain field (crack and surrounding high strain field are encapsulated inside this volume) showing how the low strain regions (<0.1% represented by navy blue colour) are located ahead of the crack tip surrounding the filler particles; this particular crack was deflected by the filler particles during its subsequent extension. Scale bar: 200 μm; false colours used in **a**–**f** to highlight features.

As shown by the 3D tomographic reconstructions in Fig. 3, the geometries of the cracks that result in the fracture of nuclear graphite are extremely complex, in particular as they interact with the pores and defects in the microstructure. Figure 3a shows a typical example of a crack in a sample fractured at 1,000 °C; for clarity, only a few filler particles are shown (marked as ‘filler’). The actual pore structure within a filler particle is shown in Fig. 3b with three different examples; it is noteworthy that some of the filler particles are not spherical and are broken into halves during manufacture (Supplementary Fig. 1). In addition to the 3D visualization of these cracks and their constituent parts, DVC analysis was undertaken. This specifically involves comparing each of the deformed 3D volumes with the reference load-free volume, so that the displacement/strain field can be deduced; further details are described in the Methods section. Before the propagation of the main crack, there is usually a zone of high strain (in excess of 0.1% when averaged over a volume of ∼100 μm^3^) formed ahead of the notch root, as highlighted in Fig. 3c. Figure 3d also demonstrates the existence of the maximum principal strain distribution (in 3D) caused by the advance of the crack as it approaches filler particles at 1,000 °C. The path followed by a growing crack is progressively more tortuous as the sample is further deformed, consistent with the highly heterogeneous distribution of the strains in 3D space associated with the significant local variations in microstructure. In addition, DVC analysis allows the strain profile across the width of the sample to be characterized. At 20 °C, nonlinear behaviour in the stress–strain curve was observed at about 90% failure load starting at a depth of 400–500 μm from the tensile surface; this depth was reduced to ∼100 μm at 1,000 °C (Supplementary Fig. 8). As reported elsewhere^20^, such nonlinear inelastic behaviour is directly associated with microcracking in this grade of Gilsocarbon graphite^21^,^22^,^23^.

### Coefficient of thermal expansion

The observed reduction in microcracks at 1,000 °C appears to be associated with the significantly higher (constrained) thermal expansion in the direction perpendicular to the graphene layers where the Mrozowski cracks are formed, that is, the excessive thermal expansion is accommodated by the closing of these cracks^11^,^12^,^13^. The coefficient of thermal expansion (CTE) of graphite single crystals, *α*, is highly anisotropic; along the *a* axis where strong *sp*^2^ hybridized C–C bonds exist within the basal planes, *α*_a_=∼−1.5 × 10^−6^ K^−1^, whereas along the *c* axis where weak Van der Waals interactions exist between the basal planes, *α*_c_=∼27.0 × 10^−6^ K^−1^. The small negative value in the basal plane corresponding to the Poisson’s contraction is associated with the large expansion in perpendicular direction. For an idealized randomly distributed set of single crystals with no porosity, the CTE at a certain temperature in any direction can be estimated by (*α*_a_+2*α*_c_)/3∼8.0 × 10^−6^ K^−1^ (refs 24, 25). The 3D quantification of the graphite microstructure provided information on the general CTE at the micro-scale in our samples when they were heated from ambient to elevated temperature. This macro-scale CTE measured at 650 °C and 1,000 °C gave a similar value of approximately 6–7 × 10^−6^ K^−1^ as an average of the 12 samples tested between 20 °C and 1,000 °C, consistent with the measured bulk properties for this grade of graphite^8^,^24^. The ∼20% reduction between our measured CTE from the tomography scans and this value is consistent with TEM observations that the lenticular Mrozowski cracks close up upon heating to accommodate the expansion perpendicular to the *c*-axis^12^ (see also Supplementary Fig. 7). This argument works on a crystallite-scale and thus would seem to be relevant only to Mrozowski cracks sized in the tens of nanometres, that is, that are contained within a single crystallite. Although the thermal expansion of individual crystallites is highly anisotropic, Gilsocarbon graphite is comprised of 70–80 wt% filler particles where the crystallite *c* axis is distributed radially outwards from the center and the Mrozowski cracks are usually radially distributed between the individual folded graphitic sheets. In the filler matrix, the distribution of these nano-pores is more random. Mrozowski cracks are found to shrink upon re-heating; indeed, they have an important role in accommodating the irradiation induced volumetric change in graphite during service^25^,^26^,^27^.

### Residual stress relaxation at temperature

**Figure 4:**
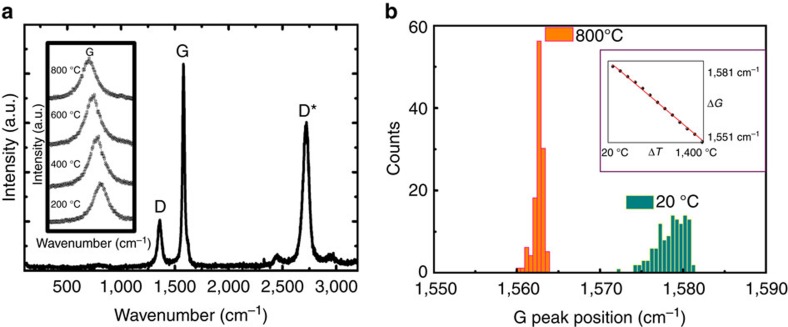
Raman spectroscopy measurements in Gilsocarbon graphite. (**a**) A Raman spectrum taken at room temperature with D and G band labelled with D corresponding to disorder activated Raman scattering and G to the *E*_2g_ Raman mode of graphite. Further higher order Raman modes such as the D* are also apparent; the insert shows the change of the G peak position with temperature up to 800 °C. (**b**) The histogram distributions of the G peak position measured in the same region at 20 and 800 °C, respectively—the inset shows the relationship between G peak position and temperature.

However, we believe that there is an additional, more important mechanism that is responsible for the increase in both strength and fracture toughness at elevated temperature in Gilsocarbon graphite; this is the existence, and subsequent relaxation, of large tensile residual stresses. To explicitly examine this in the graphite, we utilized high-temperature Raman spectroscopy to estimate the residual strain as a function of temperature. Using a 488-nm laser (2.54 eV excitation energy) Renishaw Ramascope with a hot cell, spectra from selected areas on fracture surfaces were collected at 20 °C and at 800 °C; a typical spectrum at 20 °C is shown in Fig. 4a. As described in the Methods section, the shift in the graphite G peak was measured to estimate the local residual strains retained in the crystallites within the sampled volume. Usually, the G peak position in a stress-free graphite centers around a wave number of 1,581–1,583 cm^−1^ (refs 28, 29). Shifts lower than this value represent a tensile stretch of the atomic bonds. Three regions of the graphite sample (sized 40 × 40 μm) were evaluated (as a mixture of filler particles and matrix); for each region, a map of 121 measurements (step size: 4 μm) was undertaken both at 20 °C and 800 °C, respectively. Large variations in the local residual strains were evident in the graphite crystallites at ambient temperature; however, these variations in strain mostly vanished when the sample was heated up to 800 °C. This can be appreciated from the histogram in the G peak position taken from one of the measured regions in Fig. 4b, where a wider distribution of such residual strains at 20 °C can be seen, as compared with a narrower and more uniform pattern at 800 °C. In the three mapped areas, the average of the measurements in each map gives the G peak position at 1,575, 1,577 and 1,577 cm^−1^, all with a standard deviation (s.d.) of about 2 cm^−1^ at 20 °C; however, they all shift to a value of 1,563 cm^−1^, with a s.d. about 0.7 cm^−1^, at 800 °C. Increasing the temperature leads to a linear decrease in the G peak wave number, which was calibrated to be 0.025 cm^−1^ °C^−1^ up to 900 °C in this particular graphite material. By extrapolating the Raman shifts measured at 20 °C to 800 °C, it is apparent that those measured at 800 °C result from a combination of the temperature-induced shift in phonon frequency and a relaxation of the initial tensile stretching of the atomic bonding. Such stress relaxation is also consistent with the narrower G peak position distribution (s.d.=0.7 cm^−1^ at 800 °C, compared with 2.0 cm^−1^ at 20 °C). If we take this change in the s.d. of the G peak position and convert it to a stress via the calibrated conversion factor, it represents an equivalent relaxation of tensile hydrostatic stress by ∼200 to 250 MPa when the temperature is increased from ambient to 800 °C. (As described in Supplementary Note 1, the conversion from the Raman G peak shift to the hydrostatic stress was calibrated using a diamond anvil cell where a free-standing micrometre-sized graphite and a few, 1–3 μm-sized ruby grains were subjected to a hydrostatic pressure of up to 8.9 GPa, applied by squeezing the two opposing diamond anvils, while simultaneously recording the G peak position). For a material with a macro-scale strength an order of magnitude smaller, this magnitude of residual stress of ∼200–250 MPa and its relaxation at high temperatures appears to be unrealistically large. However, as noted above, measurements on micrometre-size samples, which exclude the effects of the macro- and micro-size pores, give strength numbers for this material closer to 1,000 MPa, which, as depicted in Supplementary Fig. 2, provide, in some sense, a ‘truer’ representation of the inherent strength of the actual graphitized material^9^. The principal conclusion here is that Raman measurements indicate a significant relief of residual tensile stresses of at least 20% of the flexural strength when the graphite is heated to 800 °C. As this marked reduction in residual tensile stresses at 800 °C is also consistent with the observed (partial) closure of the Mrozowski cracks at these higher temperatures, we consider this stress relaxation to be the main contribution to the higher strength and toughness measured at elevated temperatures.

## Discussion

To confirm this residual stress relaxation argument, we performed neutron diffraction measurements on six graphite samples (with a gauge volume of 4 × 4 × 4 mm) at 20 °C; experimental details are given in Supplementary Note 2. All measurements gave a 3.375 Å average spacing between the basal planes {0002}, as compared with the standard value of 3.350 Å for a stress-free, ideal graphite hexagonal unit cell^8^,^28^,^29^, indicating that a tensile stretch in the atomic bonding is created during the manufacturing processing of Gilsocarbon graphite, consistent with the Raman observations described above. Using an elastic modulus of 34.6 GPa for this crystal lattice {0002}, an average ‘frozen-in’ tensile stress at ambient temperature can thus be calculated to be ∼260 MPa at the atomic-scale in as-manufactured Gilsocarbon graphite, that is, a residual stress value identical to the Raman spectroscopy measurements. This is considered to be a consequence of the complex manufacturing process used to make this material, with the final step involving the graphitization of the carbon material at ∼2,700 °C, or higher, to develop a larger-scale graphitic sheet structure prior to cooling from this high temperature to ambient conditions. The concomitant changes in temperature and stress state can act to cause inter-graphitic layer slip to promote the formation of complex interlayer defects, including stacking faults. Carbon interstitials, carbon complexes and vacancies, and impurity-related interstitial and substitutional defects within the crystallites, can also be formed during manufacture, as well as additional defects in the grain boundaries^30^,^31^,^32^,^33^. An overall reflection of these defects is evident in our Raman measurements in Fig. 4a by the highly pronounced intensity of the disorder-related D band (∼1,360 cm^−1^)^28^,^29^,^34^, which typically has a 0.2–0.4 intensity ratio as compared with the crystalline G band in this grade of Gilsocarbon graphite. In addition, these defects have been observed in various TEM measurements in different grades of nuclear graphites^35^,^36^. Owing to the existence of these defects, the atomic bonds are stretched. Akin to rapidly quenched martensitic structures in steels, this highly defect-ridden microstructure would be subjected to high levels of residual tensile strain, as our Raman and neutron diffraction measurements indicate, leading to initial stretching of the atom bonds in tension at ambient temperatures. In addition to the disorder-related D band in the Raman spectrum, certain studies have suggested that the double-resonance Raman mode D* at ∼2,700 cm^−1^, which is clearly in evidence in our graphite measurements (see Fig. 4a), is enhanced by the presence of stacking faults^37^. From the various studies noted above, the defects in as-manufactured graphite are clearly responsible for the tensile stretch of the atomic bonding.

Of note, as reported in ref. 38, the crystals in nuclear graphite are sometimes bonded by a non-crystalline carbon layer; as we found that these crystals are under residual tensile stresses at room temperature, it may indicate, for example, that the amorphous carbon functions as a compression counterpart. Thus, with respect to the precise length scales over which these residual stresses exist, one interpretation is that the tensile stresses exist at crystallite to grain-size dimensions, the tensile stresses being balanced by compressive stresses in the amorphous carbon surrounding these features (which naturally cannot be measured with diffraction methods or Raman spectroscopy). However, the Mrozowski cracks do not simply form at such sub-micrometre dimensions but also at dimensions up to the scale of tens to hundreds of micrometres, which suggests that the tensile residual stresses may exist over significantly larger length scales. Indeed, the overall question of the precise length scales over which the residual stresses (tensile or compressive) may exist in this highly heterogeneous material remains to be resolved, although we believe that they are associated with defects, free surfaces created by Mrozowski cracks and grain boundaries. Upon heating, these stresses will be relaxed, causing the cracks to close, to reduce their surface energy; as the material becomes correspondingly stiffer, this can affect the macro-scale fracture toughness and strength behaviour.

In summary, *in situ* mechanical testing at high temperatures was undertaken, coupled with real-time computed X-ray micro-tomography, to examine the damage evolution, strength and toughness of the nuclear Gilsocarbon graphite, which was observed to display the unusual behaviour of an increase in flexural strength and toughness with increase in temperature. Using additional Raman spectroscopy and neutron diffraction studies, we conclude that the underlying mechanisms responsible for such behaviour are the high-temperature relaxation of tensile stresses ‘frozen-in’ at ambient temperatures from the high defect concentration that results from cooling from >2,700 °C during manufacture, which aids the closure of nano-cracks at elevated temperatures from the anisotropic thermal expansion in graphite.

## Methods

### Material

The Gilsocarbon graphite material studied here is a moulded, GCMB-grade polygranular nuclear graphite used in the core of the UK AGRs manufactured by GrafTech (formerly UCAR) and supplied by EDF Energy Ltd (UK). It comprised spherical Gilsonite coke filler particles (∼500 μm in diameter) within a coal tar pitch binder matrix (density of ∼1.81 g cm^−3^ with a total porosity of 20 vol.%; macro-scale elastic modulus of about 10–11 GPa and macro-scale flexural strength of about 26–27 MPa). The vibrational moulding process used to form the graphite blocks resulted in a relative random mixture of filler and binder with an ultimate anisotropic ratio of 1:1.10 determined by comparing the macroscopic coefficients of thermal expansion between 25° and 500 °C along, and perpendicular, to the pressing direction. More detailed descriptions of the microstructure can be found in Supplementary Fig. 1.

### Mechanical testing

Two types of Gilsocarbon specimen geometries were tested. Rectangular beams of the size 3 × 3 × 16 mm and 4 × 4 × 20 mm were made by electrical discharge machining. All samples were cut longitudinally to the brick-moulding axis to ensure consistency. For strength tests, we used both 3 × 3 × 16 mm (roller span 10 mm) and 4 × 4 × 20 mm (roller span of 16 mm) geometries. Fracture toughness tests were performed on 4 × 4 × 20 mm beam samples containing a starter notch ∼1.2 mm in length and ∼400 μm in width machined using electrical discharge machining; the notch root was subsequently sharpened using a micro-notching technique involving polishing with 1 μm diamond paste using a razor blade to achieve a ∼30 μm radius at the notch tip. During this process, the crack length at both side surfaces was checked regularly to ensure a uniform front, which was later confirmed by the tomography images. Final crack lengths (notch plus micro-notch) were ∼1.6 mm, giving a crack length to width (*a/W*) ratio of ∼0.4.

For mechanical testing on the synchrotron beamline, a high-temperature test cell (hot cell) with an integrated three-point bending fixture (Supplementary Fig. 3) was used on the micro-tomography beamline 8.3.2 at the Advanced Light Source (Lawrence Berkeley National Laboratory, Berkeley, USA). Detailed descriptions of the hot cell and set-up are described elsewhere^39^,^40^. Four notched samples and four unnotched samples were tested at each temperature of 20, 650 and 1,000 °C; monotonic loading was applied to all samples at a rate of 1 μm s^−1^. The tomography scans for each sample were taken at a load-free condition (usually a small load ∼5 N was applied to make sure the sample and the rollers were in good contact to eliminate sample movement during the rotation of the load cell in the scanning process as the sample was deformed to higher load). For strength tests, several scans (six to ten) were performed from load-free condition until 85–95% of failure load was reached for DVC analysis. For fracture tests, four to five scans were made until the onset of crack growth; this was subsequently followed by up to ten scans to capture the crack extension as the sample was further loaded. After each crack growth step, the crack length was measured by averaging 40 points equally spaced across the sample thickness. An X-ray accelerating voltage of 25 keV was used for all the scans; the field of view was 4 × 4 mm at a resolution of 3.25 μm per voxel. For each scan, a set of calibration X-ray images were taken including bright-field (with the sample removed from the X-ray path) and dark-field images scan (with the X-ray blocked to correct for any stray lights); 1,025 radiographs were collected during a scan over a rotation of 180°. The information extracted from these scans, such as the crack length, was used to calculate the *J-*integral as a function of the crack extension at the different test temperatures.

For verification, additional three-point bending tests using the same rollers and spacing as the beamline experiments were performed on an Instron 5944 screw-driven loading machine (Norwood, MA, USA). These tests involving five samples each for strength and fracture toughness measurement were carried out at ambient temperature on 4 × 4 × 20 mm samples. Results were calculated using identical methods as employed for the beamline tests; values were included in the determination of the average strength in Fig. 1a and toughness in Fig. 1b. In addition, six previously dried samples, pre-exposed at 150 °C to 200 °C to remove any absorbed water, were evaluated with respect to their flexural strength. Resulting strength measurements displayed no appreciable difference from those not subjected to such thermal pre-exposure, indicating that absorbed water in the graphite had little to no effect on the mechanical behaviour of the material in the present study.

### Raman spectroscopy measurements

A Renishaw inVia Raman Microscope (Renishaw, Wotton-under-Edge, UK) with a hot cell (Linkham TS1500) was employed for this part of the study. Specifically, a 488-nm laser (LaserPhysics Model 150M-Select), corresponding to a 2.539 eV excitation energy was used to excite the Raman spectra. Calibration of the spectrometer was undertaken with silicon before and after the measurements on the graphite, considering its 520 cm^−1^ Raman line. The laser was focused onto the graphite sample surface using a Nikon long distance × 50 objective, numerical aperture=0.6, which provided a laser spot size of ∼1–2 μm. Three random areas (40 × 40 μm each) on the graphite sample surface were selected to conduct Raman mapping. For each region, a map of 121 measurements was undertaken with a step size of 4 μm at both room temperature and at 800 °C (inert argon gas was passed through the hot cell during the high-temperature measurements). A full spectrum from 0 to 3,200 cm^−1^ was acquired during each measurement with an acquisition time of 10 s. As graphite has a pseudo-hexagonal structure with the crystalline peak at 1,581–1,583 cm^−1^ (*E*_2g_ mode, G peak), a change in the G peak shift was used to estimate the residual strains in the crystallites within the sampled volume. The spectra collected from each region were then fitted using mixed Lorentzian and Gaussian using a least-square approach to derive the G peak position. Using the same hot cell and set-up, on a freestanding cylindrical piece of Gilsocarbon graphite (∼100 μm^3^ in volume containing a free-surface of a broken filler particle to focus the laser on), the G peak position was measured from 20° to 1,400 °C at intervals of 100 °C to derive the relationship between the G peak position and temperature.

### Data analysis

For calculation of strength, the geometry of each specimen was documented by optical microscopy, with dimensions confirmed, where appropriate, by the X-ray tomography reconstructed images. Flexural strength, *σ*_*f*_, values were calculated using the following solution:


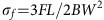


where *F* is the applied load, *L* is the loading span (either 10 or 16 mm in the present work), *W* is the sample width (either 3 or 4 mm) and *B* is the sample thickness (3 or 4 mm). The flexural modulus *E* was estimated using:


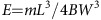


where *m* is the initial linear slope of the load-deflection curve. For specimens tested on beamline, the flexural strength and modulus were validated against the strain on the bending surface derived by DVC.

When calculating fracture toughness, nonlinear-elastic fracture mechanics methodologies were used to account for both the linear and nonlinear deformation of the graphite material in general accordance with ASTM Standard E1820 for fracture toughness testing^41^. For each data point, the value of the *J*-integral, *J*_*i*_, was determined using the solution for the single-edge-notched three-point bending geometry^10^:


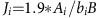


where *A*_*i*_ is the entire area under the load-deflection curve as a sum of the elastic and plastic contribution, *b*_*i*_ is the residual ligament width at a particular crack length, *a*_*i*_. Specifically, for the current work, the crack length at a certain load, *a*_*i*_, was averaged from 24 measurements on the tomography images spaced at fixed intervals across the thickness of the sample.

To express the results in terms of stress-intensity factors, the value of *J* can be converted to approximate *K* values using the mode I *J*-*K*_I_ equivalence relationship:


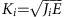


where *E* is the bulk elastic modulus, taken here to be 11 GPa for Gilsocarbon graphite^8^,^20^. There are historical bend test data showing that there can be slight change (∼10% increase at 1,000 °C (ref. 15)) in the value of *E* with temperature; however, for simplicity here, we assume an *E* of 11 GPa to convert our *J*_R_(Δ*a*) resistance-curve results to *K*_R_(Δ*a*) curves, as shown in Supplementary Fig. 4.

Finally, for visualization and volume correlation analysis, tomographic projections, that is, sinograms, were analysed using a filtered back-projection algorithm to reconstruct the tomography slices using the 8.3.2 beamline software package Octopus 8.5. The reconstructed tomographic images were then processed in ImageJ (Rasband, W.S., ImageJ, U.S. National Institutes of Health, Bethesda, http://imagej.nih.gov/ij/) and analysed in Avizo 9.2.0 (VSG, Visualization Sciences Group, Inc., Burlington, MA, USA). Segmentation was undertaken by using multiple image intensity thresholding in ImageJ and Avizo 9.2.0. DVC was undertaken using LaVision DaVis 8.3.0 software. A correlation window size of 128 × 128 × 128 (equivalent to a 104 μm^3^ volume in the real sample) was used with 75% overlap with two passes performed. Rigid body movement (including translation and rotation caused by the movement of the loading rigs) was extracted from the final results. The displacement field and the strain calculated from the DVC analysis was then exported as ‘Image raw data float (RAF)’ file and visualized using Avizo 9.2.0 software.

### Data availability

The data that support the findings of this study are available from one of the corresponding authors, Dr D.L., at Oxford University, upon reasonable request.

## Additional information

**How to cite this article:** Liu, D. *et al*. Damage tolerance of nuclear graphite at elevated temperatures. *Nat. Commun.* **8,** 15942 doi: 10.1038/ncomms15942 (2017).

**Publisher’s note**: Springer Nature remains neutral with regard to jurisdictional claims in published maps and institutional affiliations.

## Supplementary Material

Supplementary Information
