# Supplementary material for: Damage tolerance of nuclear graphite at elevated temperatures
Source: Nat Commun. 2017 Jun 30;8:15942. doi: 10.1038/ncomms15942 (PMC5497056; doi:10.1038/ncomms15942)
Supplement: Supplementary Information [file ncomms15942-s1.pdf]

Title of file for HTML: Supplementary Information
Description: Supplementary Figures, Supplementary Notes and Supplementary References

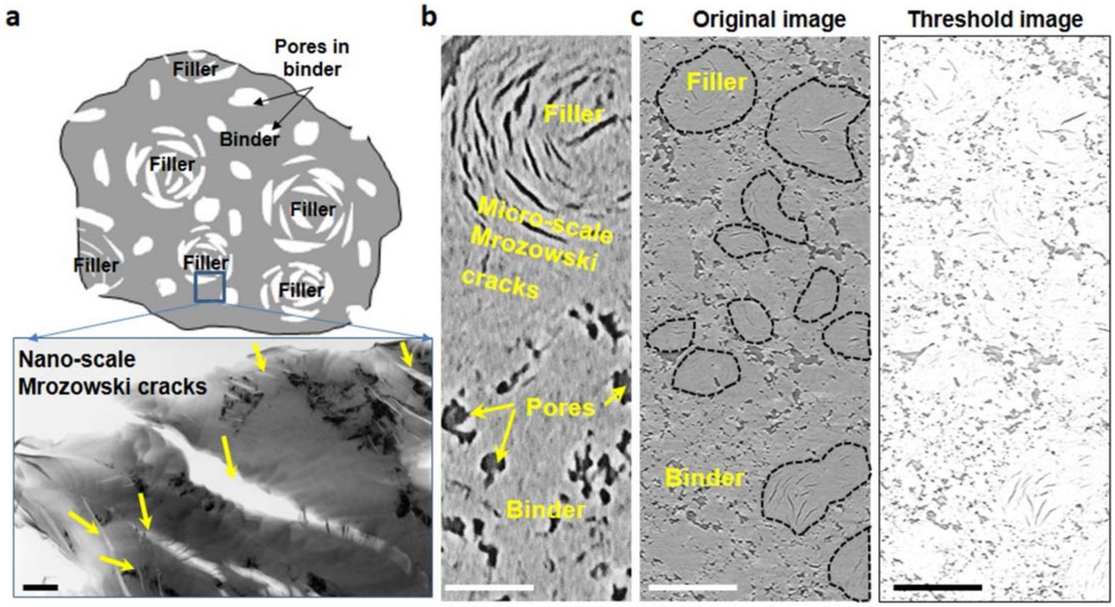

Supplementary Figure 1. 2-D representation of the multi-scale structure of Gilsocarbon graphite. (a) Schematic (representing ~1 x 1 mm area) of the distribution of filler particles (labeled as "filler") and binder phase (labeled as "binder") with the white color representing porosity/cracks. Typically, lenticular crack-like pores (also known as micro-scale Mrozowski cracks formed during cooling from calcination temperature (~1300°C) and/or graphitization temperature (>2700°C) are formed in the filler particles and lie parallel to the basal planes as a consequence of the anisotropic thermal expansion along these planes (*a*-axis) and perpendicular to it (*c*-axis)[1,2,3]. In this grade of Gilsocarbon graphite, the filler particles have a crystallite *c*-axis that is distributed radially outwards from the particle center, and thus the Mrozowski cracks appear radially distributed between the individual folded graphitic sheets (micro-size Mrozowski cracks in the filler particle are shown in the schematic). Usually spherical pores form in the binder; they are typically pore-runs resulting from the bubble percolation of volatile gases during the baking stage (~ 800°C), and micrometer-sized pores formed during the impregnation of liquid pitch. In addition to micro-size pores in the schematic, nano-size Mrozowski cracks can be viewed using transmission electrons microscopy (TEM) in both the filler particles and binder; as shown the inset, the size of these cracks covers a large range from nanometer to micrometers (the empty pores are white in color with some marked by arrows). The binder tends to have less such cracks compared with the filler particles[4]. Both nano- and micro-scale Mrozowski cracks close up upon re-heating, and thus can play an important role in accommodating the irradiation-induced volumetric change in graphite in service[5,6]. The exact volume of these nano-cracks is not easy to estimate due to the small area of observation in TEM studies. (b) A 2-D x-ray micro-tomography image (resolution 3.25 μm/pixel), showing these two types of pores in Gilsocarbon graphite described in (a,b): lenticular micro-size Mrozowski cracks distributed circumferentially in a filler particle, and the circular pores randomly dispersed in the binder phase. (c) A larger area showing the random distribution of fillers (some of them are circled by dashed lines) in the binder phase; it is this random mix of filler and binder that results in relatively isotropic bulk properties in Gilsocarbon graphite. A threshold image of the original picture in (c) highlights the pore distribution and structure in this graphite. During manufacture, about 30 parts of binder are usually used with every 100 parts of filler particles (by wt.). They are hot-mixed when the binder is in a liquid form (usually at ~100-150°C depending on the pitch type in the binder) ideally to ensure every one of the filler particles is coated with a layer of binder and is bonded together to form a macro-size graphite block. A relatively uniform distribution of filler particles is achieved in Gilsocarbon graphite by vibrational moulding. As a result, when the strength or coefficient of thermal expansion is measured in samples machined along *vs*. normal to the moulding direction, an anisotropy ratio of 1:1.10 is generally seen, indicating that the macroscopic Gilsocarbon material is effectively isotropic with respect to mechanical properties such as elastic modulus (10-11 GPa) and flexural strength (~26 MPa)[7,8,9]. More detailed descriptions of the porous structure of graphite can be found in elsewhere[10,11,12,13].

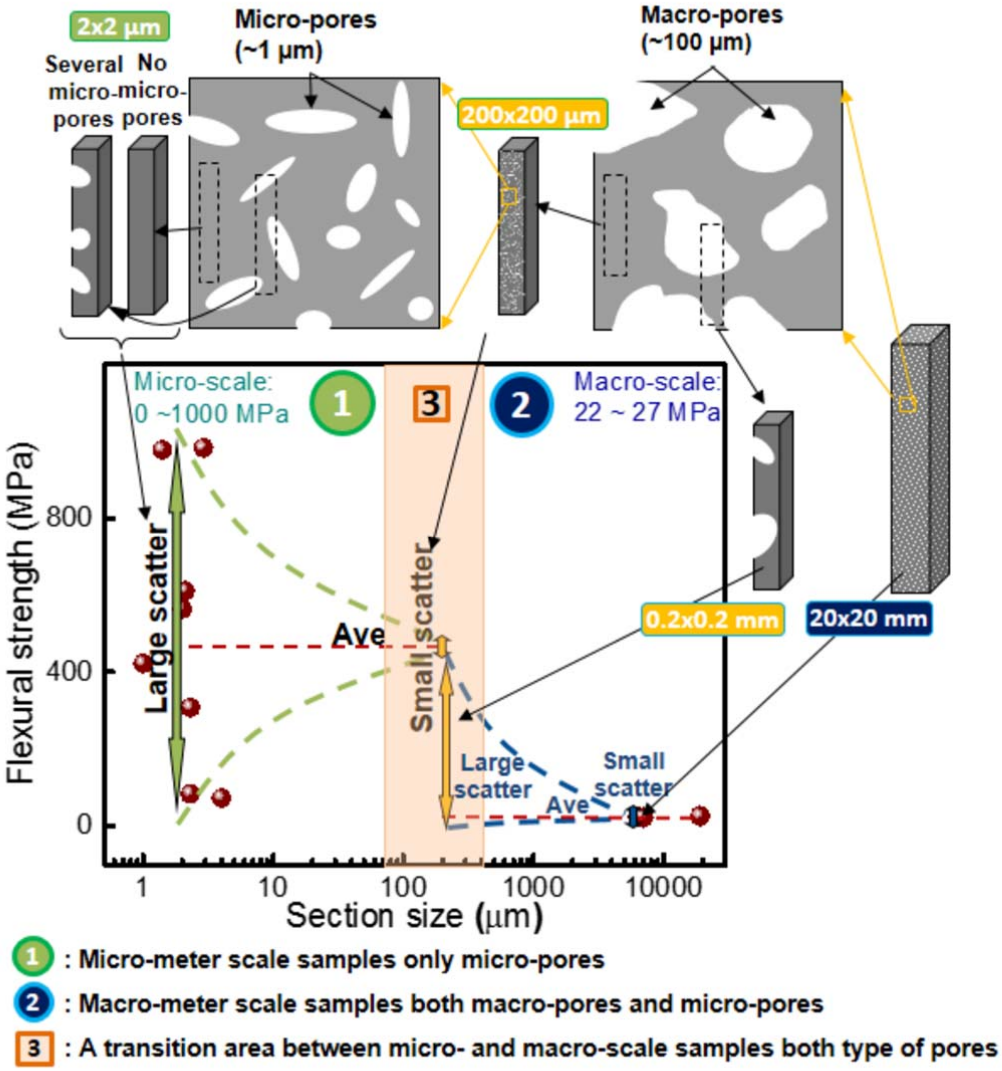

Supplementary Figure 2. Marked change in measured strength with sample section size in Gilsocarbon graphite. Due to its extensive, multiple-scale, defect population, the measured strength of Gilsocarbon can vary significantly with specimen size. At the micro-scale, micro-cantilever specimens, with a section size of typically 1 x 1 µm to 5 x 5 µm, statistically sample only a small number of micro-sized pores due to their small size, such that the measured properties depend strongly on the dimensions and the distribution of the defects in the cantilevers. The highest strength measured represents the lower bound of the matrix strength while the lowest values are from samples with detrimental pores. As the size of the specimen is increased, this permits a sufficient number of micro-pores to be sampled, with the result that the scatter in the measured strengths is reduced to an average and representative value, labelled as region 1. At the macro-scale, where the specimen dimensions are further increased (in region 2), the measured strength tends to a lower average value with less scatter. Indeed, as the larger specimens tend to sample a much greater extent of the defect population, (macro-scale) strength levels of 22-27 MPa have been measured for this material, based on some 120 samples with section-sizes ranging from 4 x 4 mm to 70 x 70 mm[14,15,7]. Note that there are two types of Gen IV VHTR that use a graphite-moderated nuclear reactor core: one is a ‘prismatic block’ and the other is a ‘pebble-bed’ core. The planned graphite for these designs is similar to Gilsocarbon in terms of the isotropic properties, but they are finer grained. However, these graphites have the same crystal structure as Gilsocarbon, and so the fundamental effects of deformation and irradiation, etc., are the same; in addition, having models that can deal with different types of graphite mesostructures makes them more robust.

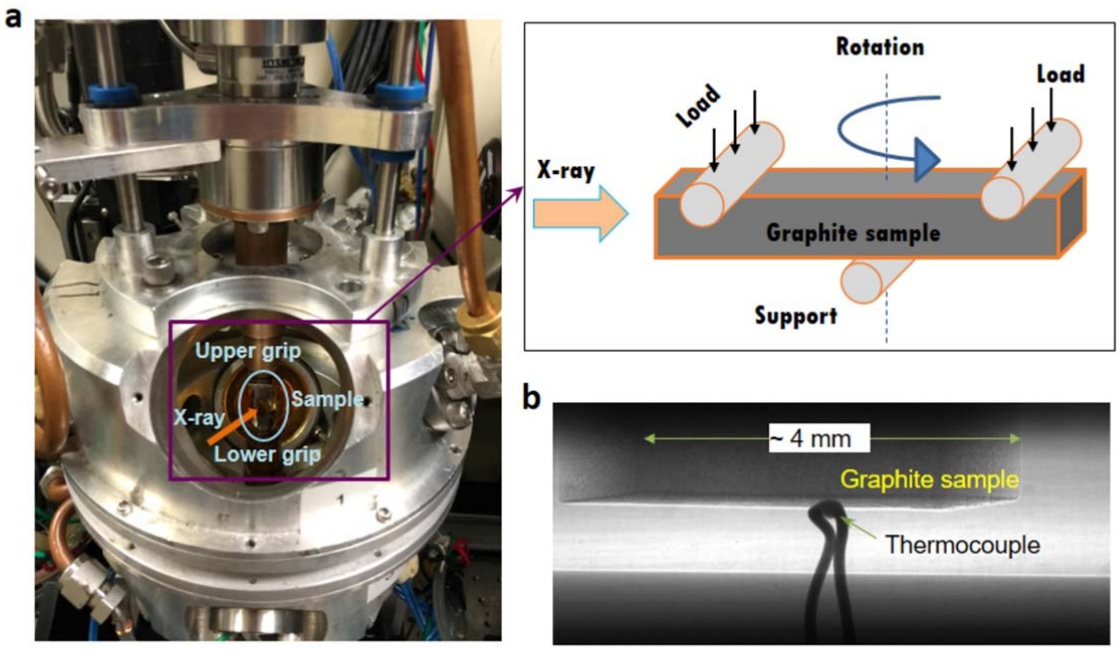

Supplementary Figure 3. Experimental setup for *in situ* high-temperature bend testing with simultaneous synchrotron x-ray micro-tomography. (a) Photograph of the hot-cell chamber used for *in situ* tomography during the measurement of strength and R-curve fracture toughness properties at both ambient and high-temperatures. A graphite three-point bend sample can be seen in place with the setup, grips, and x-ray incident route through an aluminum window. Strength and fracture toughness samples were of identical dimensions (4x4x20 mm); the fracture toughness samples additionally contained a notch of ~1.8 mm in depth. The insert is a schematic of the mechanism for the *in situ* loading combined with x-ray tomography. Due to the low attenuation of graphite to synchrotron x-ray radiation, the longer side of the sample can also be imaged with a transmission of about 50% in this particular setup; this is essential as the sample must be rotated in the x-ray beam to obtain the tomographic images. (b) A radiograph shows the placement of a thermocouple in contact with the lower side of the sample during heating. Simultaneous radiograph imaging was used as an important means to ensure the alignment of the sample during the test. Further details of this experimental synchrotron high-temperature mechanical testing facility are described elsewhere[16,17].

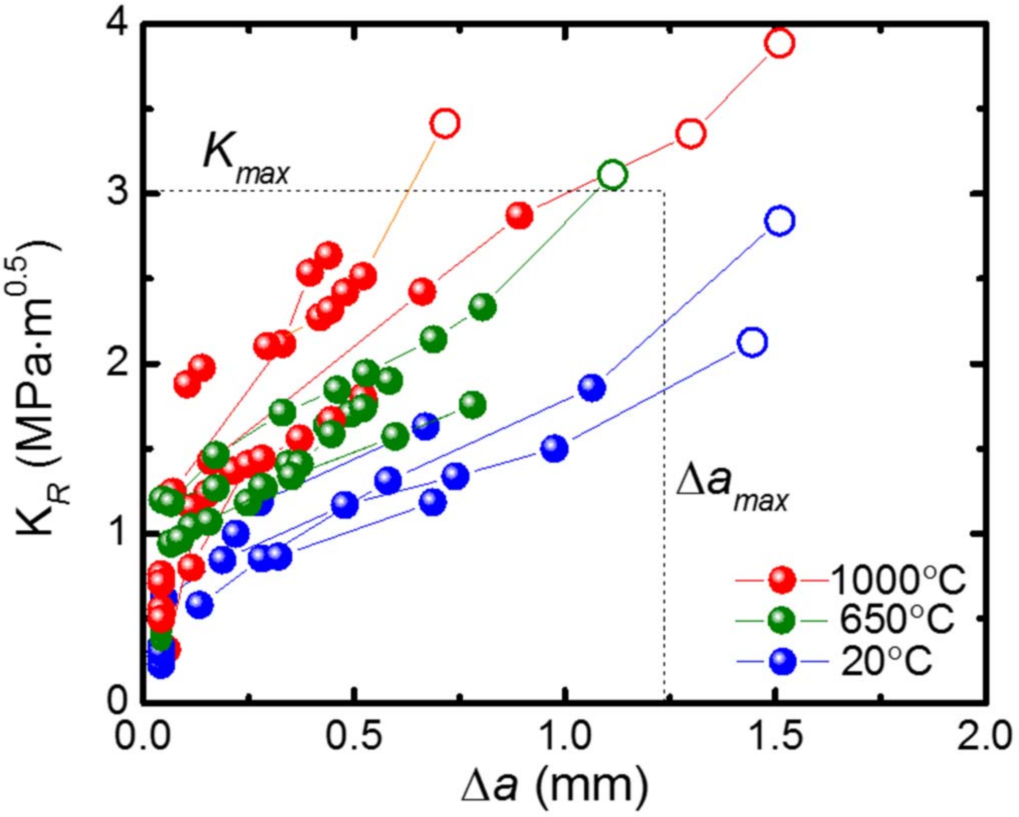

Supplementary Figure 4. Fracture toughness R-curves for this grade of Gilsocarbon graphite as a function of temperature between ambient and 1000°C, expressed in terms of stress-intensity factors. Fracture toughness R-curves are measured using nonlinear-elastic fracture mechanics in terms of J, as discussed in the main text and shown in Fig. 1b. These data can be converted into approximate stress-intensity-based $K_R(\Delta a)$ crack-resistance curves, as shown here, using the Mode I J-K equivalence of $J = K^2/E'$, where E' is the appropriate elastic modulus for plane strain, $E/(1 - \nu^2)$, where E is Young's modulus, taken here to be 11 GPa and ν is Poisson's ratio, equal to 0.22 for Gilsocarbon graphite[18]. (Note that the open data points on the R-curves are outside the maximum K and Δa limits for the size of our specimens, as prescribed by the ASTM E1820 standard[5]; these data points are therefore not included in the analysis). The K_{Ic} values are consistent with reported room-temperature values in several nuclear grade graphites[19,20,21,22,23,24,25,26].

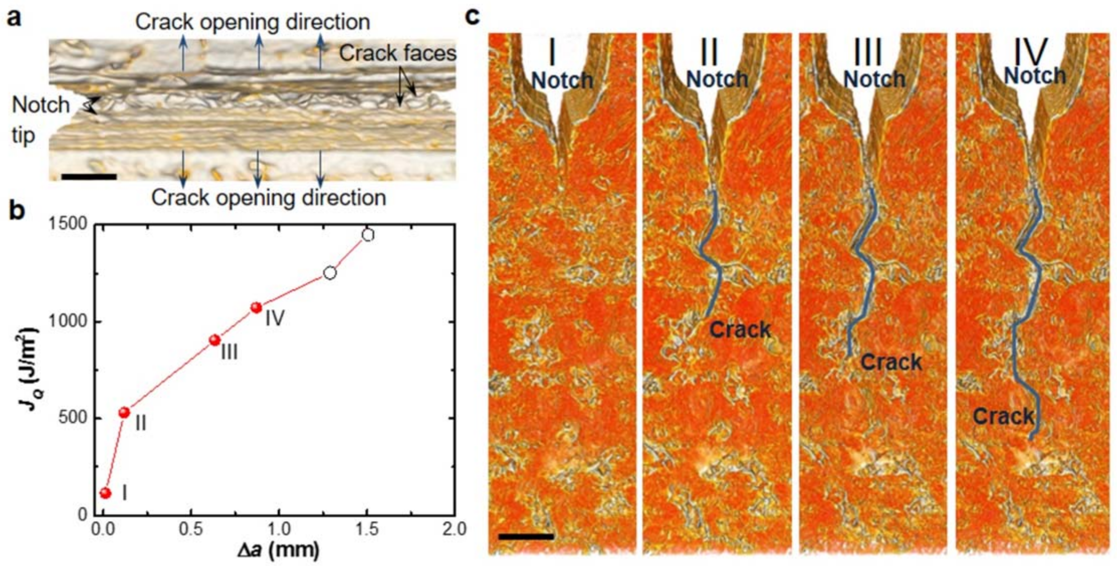

Supplementary Figure 5. Crack propagation under applied load of a sample at 1000°C. (a) Reconstructed 3-D tomographic volume showing the view from above the notch root (crack opening direction is indicated by arrows; scale bar: 100 µm). It can be seen that the inside faces of the crack are not flat but with a rather undulating feature with concave and convex regions coupled with each other. These uneven crack faces increase the free surface area as the crack propagates, thereby enhancing the energy release rate. (b) The R-curve with (c) corresponding images of the crack path as it interacts with the microstructure of the graphite during the subcritical extension of the crack. False colors have been used to delineate the crack surfaces and filler particles with the graphite; scale bar: 200 µm.

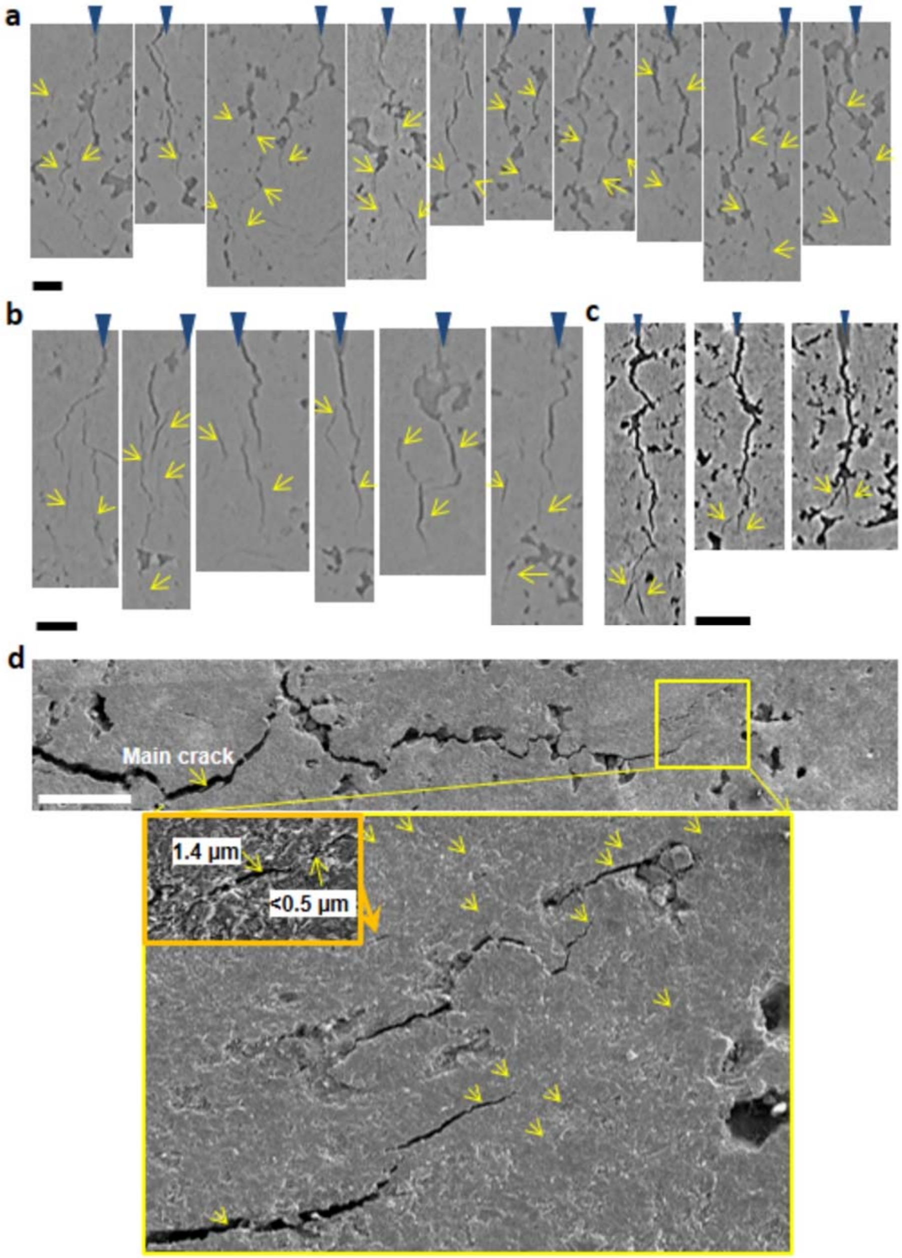

Supplementary Figure 6. Micro-cracks in Gilsocarbon graphite at 20°C. (a) In the binder matrix, the microcracks usually form ahead of, or parallel to, the main crack (marked by arrows). Some microcracks are very fine with a width comparable to the tomographic pixel resolution (3.25 µm); they tend to be discontinuous and link up the existing pores in the binder (scale bar: 100 µm). (b) In regions around/inside a filler particle, microcracks tend to form along the direction of existing lenticular shaped pores or regions between them. However, the exact density of the microcracking is difficult to quantify as the microcracks are mixed with the existing pores and defects, and many of the smaller ones are below the 3.25 µm spatial resolution of the tomography configuration (scale bar: 100 µm). (c) The microcrack density though appears to be a function of temperature; samples tested at 1000°C displayed less evidence of microcracking compared to that seen at ambient temperatures. In general, microcracks at 20°C are larger in number and finer in width, whereas at high temperatures there is more evidence of crack bridging and bifurcation, as marked by the arrows (scale bar: 100 µm). (d) Scanning electron micrograph of the crack path in Gilsocarbon graphite with images of the distributed microcracks (with various widths - micrometer and sub-micrometer sized cracks marked by arrows) that are formed ahead of the main crack tip (scale bar: 500 µm).

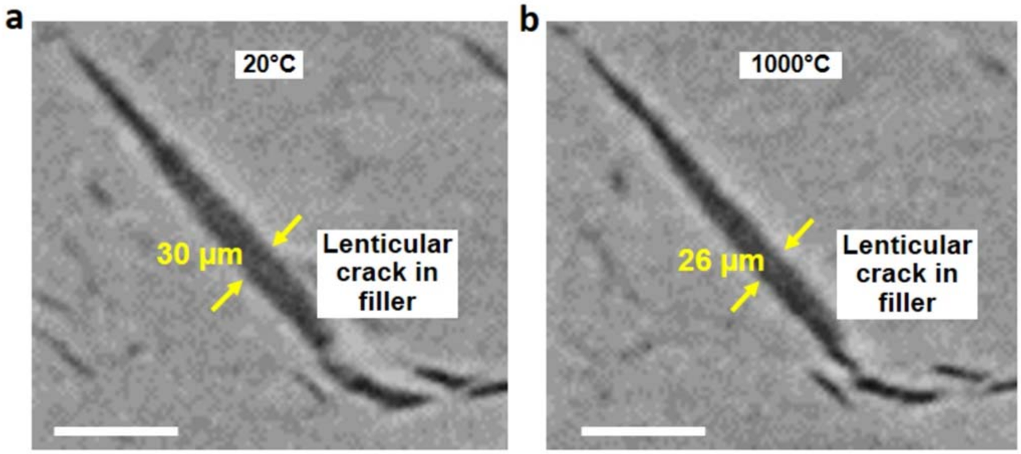

Supplementary Figure 7. Closure of lenticular cracks in filler particles in Gilsocarbon graphite during heating from ambient to high temperatures. (a) A typical lenticular crack in a filler particle at room temperature with a width of ~30 µm; (b) the same crack at 1000°C displaying a narrower width of ~26 µm. The resolution of the tomography is 3.25 µm/pixel (scale bar: 100 µm).

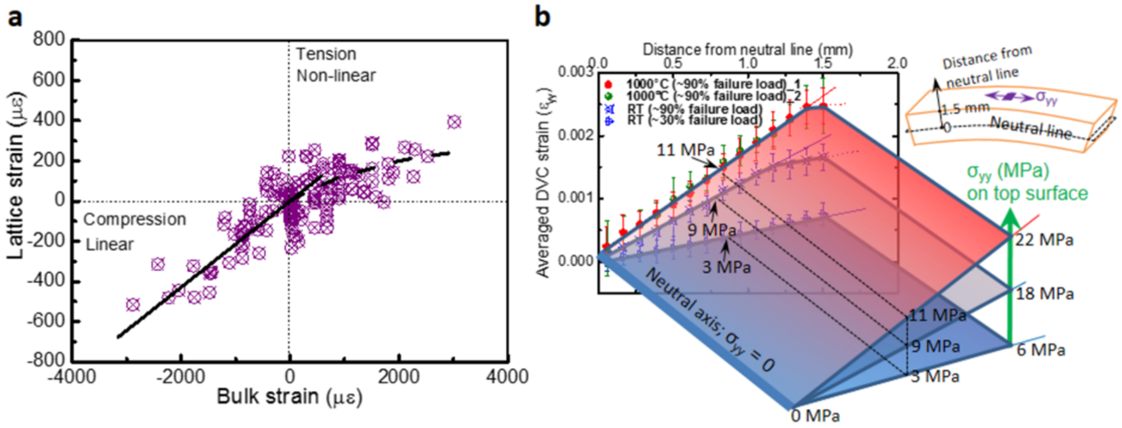

Supplementary Figure 8. **Microcracking induced nonlinear inelastic deformation behavior in bending in Gilsocarbon graphite.** (a) Neutron diffraction measurements of the change in {0002} lattice strain as a function of local bulk strain in Gilsocarbon graphite, showing that the effect of micro-cracking leads to nonlinear inelastic deformation behavior in bending at room temperature[7]. (b) To compare with the neutron and x-ray diffraction data in ref. 7, digital volume correlation (DVC) calculations of three-point bending beam samples in the present study were performed to obtain the change of tensile strain (ε_{yy}, see inset) as a function of the distance from the neutral axis. The ε_{yy} DVC strains were then projected to a third axis to correlate with the actual stress level at that load condition (σ_{yy}). This stress is calculated from beam theory assuming an elastic modulus of 11 GPa, and a linear change from the neutral axis (σ_{yy}= 0) to the top tensile surface (maximum σ_{yy}). The DVC calculation was performed as follows: the top half of a bend sample was divided up to 15 layers with each layer 100 µm thick for a 3-mm square sized specimen. The tensile strain, σ_{yy}, of each layer was averaged over the middle region (100 x100 x 100 µm) as it represents the volume subjected to the largest tensile strain in the bending geometry. Three specimens (one at 20°C, two at 1000°C) were analysed when the load was at ~90% of the failure load, and each compared with the reference volume at a load-free condition. At room temperature at low load (30% failure load, the gradient of the DVC strain was linear from the neutral line to the top surface. At high load (~90% failure load), the tensile strain ceased to increase at a depth of ~400-500 µm from the tensile surface. This shows a similar trend to that in (a), indicating the occurrence of microcracking in these regions as observed in neutron diffraction experiments in ref. 7. At 1000°C, when loaded to ~90% of the failure strain, the two samples showed similar behavior in ε_{yy} with a linear increase followed by inelastic deformation causing the nonlinearity occurring at a depth of ~100 µm below the tensile surface. This is consistent with the tomography visualization analysis that less microcracks are in evidence at high temperatures. In addition, samples at 1000°C showed a higher rate of increase in ε_{yy}, with the samples failing at a higher strain of ~0.25% compared with that of the room temperature samples (< 0.2%).

Supplementary Note 1: Calibration of residual stress measurements made using Raman spectroscopy

Raman spectroscopy was used to estimate micro-scale residual stresses by determining a calibration of the Raman shift change with a known applied pressure. Specifically, a micro-size graphite disk specimen (80 x 30 µm) was loaded into a chamber created by drilling a cylindrical hole, 150 µm in diameter, in a stainless-steel gasket pre-indented to ~50 µm thickness and mounted between two diamond anvils. A mixture of methanol and ethanol, in the proportion 4:1 by volume, and a few ruby grains, ~1 to 3 µm in diameter, were added into this chamber. The two flat diamond anvils were then squeezed close together so that a hydrostatic pressure was applied to the graphite sample. The methanol: ethanol liquid mixture provides a media to sustain a hydrostatic pressure in the chamber up to 8.9 GPa, and the ruby grains act as the indicator of the actual pressure applied. As the pressure was increased in the sample chamber, a 488-nm laser was focused through the diamond on the graphite sample and subsequently on the ruby grains. The photoluminescence from the ruby grains was collected to permit definition of the chamber pressure, specifically by converting the R2 peak shift from the stress-free position by multiplying with 7.61 $cm^{-1}.GPa^{-1}$; as such, the G peak Raman shift was measured from the graphite sample. At each loading step, the hydrostatic pressure was determined from the ruby grains before and after Raman measurements on the graphite sample; these two values were then averaged to represent the chamber pressure. Based on these procedures, a calibration of the applied hydrostatic pressure as a function of the G peak shift was obtained, which provided a pressure coefficient of ~180 ± 5 MPa/cm^{-1}. This conversion factor was used to estimate the magnitude of the relaxed stress when the sample was heated up to 800°C.

Supplementary Note 2: Neutron diffraction measurements

Neutron diffraction was used to measure the lattice spacing in this nuclear-grade Gilsocarbon graphite. The acquired measurements were then compared with stress-free single crystal graphite to estimate the residual stress-state. Six Gilsocarbon graphite samples were examined in an experiment (run number: RB1610238) performed using a time-of-flight (TOF) neutron diffractometer, ENGIN-X, with an ISIS moderator at the Rutherford Appleton Laboratory (U.K.). The primary flight path, L_1, for the neutron beam is ~50 m for the minimization of counting times; the secondary flight path, L_2, which is the distance between the instrumental gauge volume inside of the sample and the detectors, is ~1.53 m. ENGIN-X has two detector banks (Bank 1 and Bank 2) centred at $2\theta = 90°$ to the incident beam; each detector bank covers ±16° in the horizontal plane and ±21° in the vertical plane, and is made up of 5 units stacked vertically, which each unit consisting of 240 scintillators. Each scintillator is 196 mm high by 3 mm wide to provide a horizontal angular resolution of ~0.002 radians. The TOF spectra recorded by each detector is transformed into a common d-spacing scale and added together in a single diffraction spectrum via an electronic time focusing process[27,28]. The frequency of the neutron pulse is controlled by two sets of disk choppers located at 6 and 9 m from the moderator; specifically, for the present experiments, ENGIN-X was operated at 25 Hz. The specimen was placed in the middle of the sample stage at a 45° angle with respect to each detector. The lattice spacing in two directions, $d_{45°}$ and $d_{-45°}$, was measured along the direction of the impulse exchange vector, respectively. Under elastic collision conditions, the wavelength of the detected neutrons, λ, is correlated from its time of flight by:

$$\lambda = \frac{h}{m(L_1+L_2)} t \, , \qquad (1)$$

where h is the Planck's constant, t is the TOF, m is the neutron mass, L_1 and L_2 are the distance primary and secondary flight paths, respectively. Each peak in the resultant neutron spectrum corresponds to a $\{hkl\}$ family of lattice planes; according to Bragg's law, the *d*-spacing of the lattice plane can be derived from the position of the corresponding peak:

$$d_{hkl} = \frac{h}{2sin\theta m(L_1+L_2)} t_{hkl} \quad , \qquad (2)$$

where θ is the half angle between the incident neutron beam and the scattered path. In our diffraction measurements, the peak position was determined by fitting an individual peak using a least-squares refinement of individual peaks. In the present work, only the (0002) peak and lattice spacing were analysed. The instrumental gauge volume along the beam was defined using radial collimators and five sets of removable radial collimators providing 0.5, 1, 2, 3 and 4 mm gauge width options. The 4-mm gauge was chosen with a 20 mins acquisition time for the current tests to reduce count times and keep the background low by eliminating neutrons scattered from sample-environment equipment. Prior to each measurement on the graphite, CeO_2 powder in a vanadium tube was used to calibrate the time-of-flight to *d*-spacing function. In addition, a vanadium rod was used to calibrate the peak height by collecting an incident wave spectrum. Details of the calibration regarding the ENGIN-X instrument can be found elsewhere[29]. The measurements from the six samples, 12 data points (6 data from each of the two detectors that collects lattice spacing information from orthogonal directions) all gave an identical {0002} lattice spacing of 3.375 Å. As there was no appreciable difference between the data acquired from the two detectors, the 4 x 4 mm gauge volume displayed a sufficient number of crystallites within the filler particles and binder matrix to provide a representative evaluation of the lattice parameter.

Supplementary References

1. Mrozowski, S. Anisotropy of thermal expansion and internal stresses in polycrystalline graphite and carbons. *Phys. Rev.* **86,** 622–622 (1952).
2. Karthik, C., Kane, J., Butt, D. P., Windes, W. E. & Ubic, R. Microstructural Characterization of Next Generation Nuclear Graphites. *Microsc. Microanal.* **18,** 272–278 (2012).
3. Hacker, P. J., Neighbour, G. B. & McEnaney, B. The coefficient of thermal expansion of nuclear graphite with increasing thermal oxidation. *J. Phys. D. Appl. Phys.* **33,** 991–998 (2000).
4. Wen, K., Marrow, J. & Marsden, B. Microcracks in nuclear graphite and highly oriented pyrolytic graphite (HOPG). *J. Nucl. Mater.* **381,** 199–203 (2008).
5. Krishna, R., Jones, A. N., McDermott, L. & Marsden, B. Neutron irradiation damage of nuclear graphite studied by high-resolution transmission electron microscopy and Raman spectroscopy. *J. Nucl. Mater.* **467,** 557–565 (2015).
6. Burchell, T. D. & Snead, L. L. The effect of neutron irradiation damage on the properties of grade NBG-10 graphite. *J. Nucl. Mater.* **371,** 18–27 (2007).
7. Marrow, T. J. *et al.* In situ measurement of the strains within a mechanically loaded polygranular graphite. *Carbon.* **96,** 285–302 (2016).
8. Marsden, B. J. *et al.* Dimensional change, irradiation creep and thermal/mechanical property changes in nuclear graphite. *Int. Mater. Rev.* **61,** 155–182 (2016).
9. Brocklehurst, J. E. & Darby, M. I. Concerning the fracture of graphite under different test conditions. *Mater. Sci. Eng. A* **16,** 91–106 (1974).
10. Mileeva, Z., Ross, D. K. & King, S. M. A study of the porosity of nuclear graphite using small-angle neutron scattering. *Carbon.* **64,** 20–26 (2013).
11. Zhou, Z. *et al.* From nanopores to macropores: Fractal morphology of graphite. *Carbon.* **96,** 541–547 (2016).
12. Vertyagina, Y. & Marrow, T. J. Multifractal-based assessment of Gilsocarbon graphite microstructures. *Carbon.* **109,** 711–718 (2016).
13. Laudone, G. M., Gribble, C. M. & Matthews, G. P. Characterisation of the porous structure of Gilsocarbon graphite using pycnometry, cyclic porosimetry and void-network modeling. *Carbon.* **73,**

61–70 (2014).
14. Liu, D., Nakhodchi, S., Heard, P. & Flewitt, P. E. J. Small-scale Approaches to Evaluate the Mechanical Properties of Quasi-brittle Reactor Core Graphite. *Graph. Test. Nucl. Appl. Significance Test Specim. Vol. Geom. Stat. Significance Test Specim. Popul.* **STP1578,** 1–21 (2015).
15. Šavija, B. *et al.* Experimentally informed multi-scale modelling of mechanical properties of quasi-brittle nuclear graphite. *Eng. Fract. Mech.* (2015). doi:10.1016/j.engfracmech.2015.10.043
16. Bale, H. A. *et al.* Real-time quantitative imaging of failure events in materials under load at temperatures above 1,600 °C. *Nat. Mater.* **12,** 40–46 (2012).
17. Haboub, A. *et al.* Tensile testing of materials at high temperatures above 1700 °C with in situ synchrotron X-ray micro-tomography. *Rev. Sci. Instrum.* **85,** 83702 (2014).
18. Arregui-Mena, J. D., Bodel, W., Worth, R. N., Margetts, L. & Mummery, P. Spatial variability in the mechanical properties of Gilsocarbon. *Carbon.* **110,** 497–517 (2016).
19. Li, H., Li, J., Singh, G. & Fok, A. Fracture behavior of nuclear graphite NBG-18. *Carbon.* **60,** 46–56 (2013).
20. Yoon, J. H., Byun, T. S., Strizak, J. P. & Snead, L. L. Characterization of tensile strength and fracture toughness of nuclear graphite NBG-18 using subsize specimens. *J. Nucl. Mater.* **412,** 315–320 (2011).
21. Takahashi, S., Aoki, S. & Oku, T. Impact fracture toughness of a nuclear graphite measured by the one-point-bending method. *Carbon.* **31,** 315–323 (1993).
22. Hodgkins, A., Marrow, T. J., Mummery, P., Marsden, B. & Fok, A. X-Ray tomography observation of crack propagation in nuclear graphite. *Mater. Sci. Technol.* **22,** 151–1045 (2006).
23. Ouagne, P. *et al.* Crack growth resistance in nuclear graphites. *J. Phys. D. Appl. Phys.* **35,** 315 (2002).
24. Mostafavi, M., McDonald, S. A., Mummery, P. M. & Marrow, T. J. Observation and quantification of three-dimensional crack propagation in poly-granular graphite. *Eng. Fract. Mech.* **110,** 410–420 (2013).
25. Su, R. K. L. *et al.* Determination of the tension softening curve of nuclear graphites using the incremental displacement collocation method. *Carbon.* **57,** 65–78 (2013).
26. Mostafavi, M., McDonald, S. A., Çetinel, H., Mummery, P. M. & Marrow, T. J. Flexural strength and defect behaviour of polygranular graphite under different states of stress. *Carbon.* **59,** 325–336 (2013).
27. Jorgensen, J. D. *et al.* Electronically focused time-of-flight powder diffractometers at the intense pulsed neutron source. *J. Appl. Crystallogr.* **22,** 321–333 (1989).
28. Kropff, F. On the design of time-focused detector banks for pulsed neutron TOF spectroscopy. *Nucl. Instruments Methods Phys. Res. Sect. A Accel. Spectrometers, Detect. Assoc. Equip.* **245,** 125–136 (1986).
29. Santisteban, J. R., Daymond, M. R., James, J. A. & Edwards, L, ENGIN-X: A third-generation neutron strain scanner. *J. Appl. Crystallogr.* **39**, 812-825 (2006).
